# Murphy’s Law and Aesthetic Surgery: Are Outcomes Worse in Medical Doctor and Very Important Patients?

**DOI:** 10.3390/medicina60111807

**Published:** 2024-11-04

**Authors:** Nicola Zingaretti, Francesco De Francesco, Michele Riccio, Massimo Robiony, Alessandro Tel, Salvatore Sembronio, Lavinia Bucciarelli, Pier Camillo Parodi

**Affiliations:** 1 Department of Medicine (DMED), Clinic of Plastic and Reconstructive Surgery, Academic Hospital of Udine, University of Udine, 33100 Udine, Italy; 2 Department of Reconstructive Surgery and Hand Surgery, Azienda Ospedaliera Universitaria “Ospedali Riuniti”, 60126 Ancona, Italy; 3 Maxillofacial Surgery Department, Academic Hospital of Udine, Department of Medicine (DMED), University of Udine, 33100 Udine, Italy; 4 School of Psychology, University of Padova, 35131 Padova, Italy

**Keywords:** Murphy’s Law, VIP, complications, patient safety, aesthetic surgery, influential patients, entitled patients, physician patients, patient management, midface rejuvenation

## Abstract

*Background and Objectives*: Surgeons have long been aware of Murphy’s Law: “If anything can go wrong, it will”. When applied to surgery, Murphy’s Law suggests that if there is a way that an operation can be set up incorrectly then someday, somewhere, it will be set up incorrectly. This paper focuses on complications in medical doctor (MD) and VIPs during aesthetic surgery. *Materials and Methods*: We evaluated the clinical results of 368 MDs/VIPs (group 1) and 368 non-MDs/VIPs (group 2) who underwent aesthetic surgery (upper blepharoplasty, facelift, breast augmentation) between January 2010 and September 2021. The minimum follow-up after surgery was 2 years. *Results*: There was no statistically significant difference in the rate of complications between the two groups. Among the treated patients, the percentage of complications was similar to what has been reported in the literature. Interestingly, the time spent in surgery was longer, and there was an increased number of admissions to outpatient clinics in group 1. *Conclusions*: We suggest changing the current perception of Murphy’s Law regarding complications in MD patients/VIPs undergoing aesthetic surgery.

## 1. Introduction

Legend has it that Murphy’s Law was formulated in 1949 by a Captain Edward A. Murphy, then at Edwards Air Force Base in California. The first law states: “If anything can go wrong, it will”. There are several variants of Murphy’s Law, all meant to express a perverse outcome. Other notable Murphy Laws include: “Everything goes wrong all at once”, “Nature always sides with the hidden flaw”, “Things get worse under pressure” and “Every solution breeds new problems”.

Murphy’s Law certainly applies to surgery. Doctors are no more immune from Murphy’s Law than engineers, architects, builders, or even accountants. It is not a general belief but often commented on anecdotally by surgeons and operation room staff that medical doctors have more difficult operations and have a greater complication rate than nonmedical patients. But, whether the Murphy’s Law is real or imaginary remains an open question [1,2,3,4]. More specific in this field is the Segway’s First Risk Theorem, which states “The likelihood of medical errors increases exponentially in VIPs”. VIPs, in this case, are considered to be people accorded special privileges because of their status or wealth. For example, they may be royalty, celebrities, members of government, or the heads of important companies [5]. Although their special status may lead many to assume that VIPs have access to faster and better care and receive special attention from their medical providers, in actuality, their VIP status may be causing more harm than good. 

There are no data in the medical literature on aesthetic surgery outcomes in MD patients/VIPs. Given the absence of a large multicenter study in the literature, in an attempt to answer this question, the objectives of this study were to use data to (1) determine if outcomes for aesthetic surgery in a group of medical doctors and VIPs were worse than the incidence of complications in nonmedical/non-VIPs for the same intervention; (2) confirm the validity of Murphy’s Law and understand the reasons why these patients might have a different experience than other patients who undergo the same surgery.

## 2. Materials and Methods

A retrospective chart review was conducted on 368 consecutive physician patients and/or VIPs (group 1) who underwent primary facelift/upper blepharoplasty/breast augmentation for aesthetic reasons between January 2010 and September 2021. 

The control group (group 2) was set up by including the same number of consecutive patients (nonphysician patients and/or VIPs) who underwent the same operation in the same time interval. 

The exclusion criteria were preoperative dry eye syndrome, concomitant surgical interventions (such as other aesthetic procedures or interventions on the face or breast), previous surgery on face/breast, alopecia, allergies, history of aesthetic medical procedures on face/breast (not treated with hyaluronic acid/botulinum toxin or other invasive treatments in the previous year), history of coagulopathy, concurrent use of anticoagulant or hormone therapy, and high-risk patients (ASA scores of 3 and 4).

We collected and evaluated the complication rates after the operations, operation room (OR) time (in this case, determined as the total time spent in an OR exclusive of preoperative [preparation] and postoperative [recovery] time), and number of admissions to outpatient clinics for medication, and we compared these with the literature data for the same interventions.

Complications were defined as any adverse postoperative event occurring as a direct consequence of the intervention and requiring close observation or additional treatment. Before commencing this study, the ethics committee of our institution approved the study design.

### 2.1. Surgical Procedures

The same surgical technique for each intervention was used in all patients in the two groups. All surgeries were performed by the senior authors (upper blepharoplasty and facelift by P.C.P. and M.R., breast augmentation by P.C.P.).

#### 2.1.1. Upper Blepharoplasty

The surgeries were performed under local anesthesia with a 30-gauge needle using a diluted solution of 0.1% lidocaine, 1:100,000 adrenalin. On average, 10 mL of this infiltration solution was applied to each eyelid and left for at least 10 min to ensure adequate blanching of the infiltrated skin.

The technique used was as follows: the eyelid incision was marked, highlighting the excess tissue to be resected, while the patient was in a seated position looking straight ahead at a fixed point. A 0.5% chlorhexidine-alcohol solution was used to achieve antisepsis of the surgical field. An upper eyelid incision was made with a #15 blade scalpel to the highlighted section. A strip of orbicular muscle was removed from the eye, followed by dissection of the preseptal portion and fat pouches. Excess fat in the pouches was then excised and cauterized using electrocautery. A 6-0 nylon suture was used to perform intradermal continuous skin sutures. The surgical site was then cleaned with a sterile buffered saline solution. Finally, adhesive tape (Steri-strip^TM^, 3M Health care, Eden Prairie, MN, USA) was applied over the surgical wounds.

Six to eight days after surgery, the sutures were removed. The patient was reexamined only if necessary in the three months following surgery [6,7,8]. 

#### 2.1.2. Facelift

The surgeries were performed under local anesthesia with minimal sedation. Deep vein thrombosis prophylaxis in the form of thrombo-embolus-deterrent compression stockings and sequential compression devices was used for every patient.

Antibiotics were administered intravenously based on the hospital standard (2 g of cefazolin before the start of anesthesia). 

A solution of 0.5% chlorhexidine-alcohol was used to achieve antisepsis of the surgical field. Tumescent infiltration with local anesthesia was provided with a 22-gauge spinal needle using a diluted solution of 0.1% lidocaine, 1:100,000 adrenalin, and normal saline. An average of 30 mL of this infiltration solution was used on each side of the face and left for at least 10 min to ensure adequate blanching of the infiltrated skin. The initial incision using a #15 blade was made beginning at the temporal area anterior to the ear and running into the postauricular sulcus. The preauricular incision initiated at the most caudal end of the earlobe was directed upward, following the earlobe crease, crossing the incisura intertragica perpendicularly, making a little indentation to proceed to the tragal rim, further following the hairline in the non-hair-bearing recess in front of the ear, and then turning anteriorly along the inferior limit of the sideburn. The cut could be extended as needed to provide sufficient subcutaneous dissection for an acceptable vector for SMAS traction based on the individual patient’s needs. Hydrodissection was used to develop the subcutaneous tissue plane, facilitating flap elevation.

The flap was then undermined and elevated with scissors, using either a snipping or spreading technique. The superficial musculoaponeurotic system plane was identified as the fibrovascular layer deep to the subdermal fat and superficial to the parotid fascia. SMAS plication rhytidectomy to achieve the desired lift was then performed using a 2-0 quill suture; the suspension was reinforced with polydioxanone sutures. Hemostasis was meticulous to avoid hematoma formation, and a closed suction drain was placed in each side. Closure of the wound involved the suspension of the skin in an appropriate superolateral vector and excision of the excess, taking care to avoid placing too much tension across the wound. A 6-0 nylon suture was used to perform continuous skin sutures [9,10,11]. 

Patients were instructed to sleep upright for the first week to minimize edema and given a neck support dressing for the 2 weeks after surgery. Sutures were removed on postoperative day seven, and patients were seen at three and six weeks for follow-up. Thereafter, the patient was seen only if necessary in the three months following surgery.

#### 2.1.3. Breast Augmentation

The surgeries were performed under local anesthesia with sedation. A 0.5% chlorhexidine-alcohol solution was used to achieve antisepsis of the surgical field. 

Tumescent infiltration with local anesthesia was provided with a 22-gauge spinal needle using a diluted solution of 0.1% lidocaine, 1:100,000 adrenalin, and normal saline. On average, 100 mL of this infiltration solution was applied to each breast in a subcutaneous and submuscular plane and left for at least 10 min to ensure adequate blanching of the infiltrated skin [12,13,14]. 

Patients underwent submuscular augmentation mammoplasty using silicone implants with texturized or smooth coverings, as selected by the surgeon. The implants were inserted using an inframammary fold incision. The prosthesis shape and volume varied among patients on the basis of a consensus between the surgeon and the patient. A closed suction drain was placed. A 4-0 poliglecaprone suture was used to perform subdermal and intradermal points on two planes. The surgical site was then cleaned with a sterile buffered saline solution. Then, four layers of sterile cotton gauze compresses were placed side-by-side to completely cover the wound and were fixed with microporous adhesive tape.

Antibiotics were administered intravenously based on the hospital standard (2 g of cefazolin before the start of anesthesia). At the end of the operation, all patients wore postoperative bras for at least 30 days after surgery.

Six to eight days after surgery, the dressings were renewed, the wound was disinfected, and adhesive tape (Steri-strip^TM^) was applied over the surgical wounds. Fourteen days after surgery, the dressings were removed, and adhesive tape was applied over the surgical wounds for 5 days. Thereafter, the patient was seen only if necessary in the three months following surgery. 

### 2.2. Statistical Analysis

The Kolmogorov–Smirnov test revealed non-normally distributed data; therefore, all statistical analyses were carried out according to a nonparametric approach. To investigate the differences between our experience and the literature data, we calculated the percentages of all data for each group, the corresponding median values, and 95% confidence intervals. The median values were then compared using the Mann–Whitney U test. The threshold for statistical significance was set at *p* < 0.05. Repeatability was calculated as a standard deviation to determine the differences between measurements using SPSS version 16.0 software (SPSS Inc., Chicago, IL, USA).

## 3. Results

The patients’ demographics are reported in Table 1. 

All patients were of European descent. Of the 736 patients included in this study, 83% (609) were women, and 17% (127) were men, with a mean age of, respectively, 51.6 years (36 to 78) for patients who underwent upper blepharoplasty, 57.1 years (46 to 79) for patients who underwent facelift, and 30.4 years (18 to 56) for patients who underwent breast augmentation.

For the patients who underwent upper blepharoplasty, the complications found were 28 patients (13.2%) with symptomatic dry eye for group 1 and 23 (10.9%) for group 2; 2 cases (0.9%) of conjunctival chemosis for group 1 and 3 (1.4%) for group 2; 0 patients with infection and retrobulbar hemorrhage or blindness.

In the cases reviewed, surgery lasted from a minimum of 34 min to a maximum of 63 min (average of 52 min per surgery in group 1 and 44 min in group 2) for upper blepharoplasty. The follow-up period was 95 months for group 1 and 101 for group 2, with a range of 32–128 months. On average, the patients returned to the clinic 2.3 times in the 3 months following surgery for group 1 and 1.2 for group 2, with a range of 1–4 (Table 2A).

Postoperative complications after facelift included three minor hematomas (4.47%) for group 1 and five (5.97%) for group 2, one major hematoma (1.49%) in each group, and two seromas (3.77%); no patients developed infections or permanent nerve damage. One patient in group 1 and two in group 2 presented temporary nerve damage. The incidence of skin flap necrosis following facelift was 4.47% (three patients) for group 1 and 3.77% (two patients) for group 2.

In the cases reviewed, surgery lasted from a minimum of 86 min to a maximum of 129 min in group 1 (average of 106 min per surgery) for facelift; surgery lasted from a minimum of 91 min to a maximum of 116 min in group 2 (average of 99.5 min per surgery). The follow-up period was 67.2 months in group 1 (range of 30–108 months) and 89.4 in group 2 (34–128 months) (Table 2B).

One-hundred-seventy-eight pairs of prostheses were placed submuscularly using the dual plane technique. Regarding the type of prosthesis used, 132 pairs were textured prostheses (74%), while 46 pairs were smooth prostheses.

Postoperative complications after breast augmentation included three hematomas (3.37%) in each group, one seroma (1.12%) in group 1, one intracapsular implant rupture (1.12%) in group 1 and two (2.24%) for group 2. Five patients developed animation deformity (5.61%) in group 1 and four (4.49%) in group 2; eleven patients developed rippling (six in group 1 and five in group 2), seven patients capsular contracture (four in group 1 and three in group 2). Twelve patients required revision in group 1 (13.48%) and eight in group 2 (8.98%). One of the patients presented with infection. 

In the cases reviewed, surgery lasted from a minimum of 45 min to a maximum of 78 min (average of 69 min per surgery for group 1 and 56 min for group 2) for breast augmentation; the follow-up period was 58 months for group 1 and 76 for group 2, with a range of 26–118 months (Table 2C).

## 4. Discussion

Doctors and VIPs being subject to “Murphy’s Law”, with a higher incidence of adverse outcomes, is often quoted anecdotally but has never been scientifically tested in plastic surgery. Medical care may indeed be compromised for patients with uncommon social standing. This concept has been the subject of dozens of articles and reviews over the last 70 years, as discussion of the outcomes for this special cohort of patients has captured various levels of interest. Geary M. et al. noted no differences in pregnancy outcomes between medical doctors and a group of nondoctors [4]. The care of Very Important Patients (VIPs) may differ from that of other patients for a variety of reasons such as better access to resources or greater attention from health care staff. The term ‘VIP’ itself, though readily understood and used regularly in the medical literature, refers to a widely diverse group of patients. We define a Very Important Patient (VIP) as someone with the potential to significantly influence a clinician’s judgment or behavior. Whether they be doctors, celebrities, the politically powerful, or philanthropists, their social status together with their behavior may influence the approach of their medical care provider. This deviation from standard practices may result in an inferior quality of care. Gaining an understanding of what common features this widely disparate cohort of patients may share can help health care providers manage ethical concerns when they arise. 

Given our definition of VIP, it is clear that patients who are also physicians can be considered VIPs, as their presumed knowledge or position can influence the judgment of the treating physician [23].

Physicians in the position of caring for other physicians may feel conflicted in their dual roles of colleague and physician [24,25]. The treating physician may fail to fully evaluate the patient as medically indicated. In such a situation, the treating physician may further deviate from the standard of care by prescribing treatments that could be considered either too conservative or too progressive [24,25,26]. Moreover, when a physician is treating a fellow physician, they might assume that the physician patient understands or knows what and why a particular treatment is being considered, even if the patient has a completely different specialty. This may result in the treating physician mistakenly accepting the patient’s opinion [4]. Further complicating the issue is the additional knowledge and experience of physician patients, which might require extra efforts to reassure them, causing further deviation from standard practices [27]. 

Furthermore, administering care to VIPs presents its own set of challenges. In a situation often referred to as “VIP syndrome” [25,28,29,30,31], a patient’s special social or political status—or the medical care providers’ perceptions of it—can bring about changes in behaviors and clinical practices that create a “vicious circle of VIP pressure and staff withdrawal” that can lead to poor outcomes [30]. Medical staff faced with caring for VIPs may feel pressured to change their usual clinical practices. 

Although this study dealt with a relatively small number of patients, we found that there were no ‘clinically’ significant differences other than ‘statistically’ significant differences between MDs/VIPs and the rate of complications for non-medical-professional/non-VIPs. Interestingly, we noted that the operation times of our treated patients were longer than the average operation time reported in the literature. 

An explanation could be that the treatment of MDs/VIPs seems to be linked to an increase in the levels of attention of the surgeon [23,24,25,26]. This increased attention, despite the interventions being of a type performed routinely and on generally healthy patients, may be due to the complications and negative consequences that are possible as postoperative outcomes.

Performing a task under this type of pressure could therefore induce specific concerns in the surgeon, otherwise known as “performance anxiety” [32]. In this situation, the surgeon may be more inclined to perceive a sense of threat coming from the surrounding social context and above all may fear the questioning of their abilities and subsequent humiliation. In the event of a negative outcome of the surgery (in this case, the presence of complications following the surgery performed), the surgeon could in fact perceive their personal and professional identity as being in danger [32].

This state of mind would encourage a prudent attitude on the part of the surgeon, which may take the form of examining a significant number of preoperative surgical options and carrying out continuous checks during surgery and numerous postoperative visits. As a consequence, time spent in surgery may become somewhat extended, as the results obtained in our cases demonstrate.

Although such prudence, when implemented in a balanced way, may positively affect the motivation of the surgeon and improve their performance, an excess of caution may, on the contrary, lead to high levels of stress in the surgeon.

The surgeon’s willingness to review these patients several times, due to their social status, could also distort the normal doctor–patient relationship and influence the surgeon’s leadership, consequently having a negative influence on the overall operative outcome [32].

There has been some positive evidence on the effectiveness of mindfulness meditation techniques [33,34,35] in countering the high levels of stress in specialist surgery. In this context, the mindfulness-based stress reduction (MBSR) protocol is interesting: a stress reduction program based on mindfulness and aimed at improving well-being, relaxation states, and related positive emotions [35]. This would allow the surgeon to restore a level of “arousal” appropriate to the specific context, since mindfulness training is positively linked to resilience, well-being, and the improvement in executive functions and performance [33].

Brief mindfulness sessions may help a surgeon address stress before surgery [33], such as those offered by Surgical Mindfulness (an online platform available for both iOS and Android devices). This app designed for surgeons and operating room personnel includes preoperative meditation, breathing techniques, a hand wash timer, and access to the Mindful Attention Awareness Scale (MAAS).

On the basis of our observational data, we now describe the following suggested procedures that a surgeon should apply when they have a physician patient or VIP:

1. Set limits and perform surgery equally for all patients.
2. Stay grounded.
3. Maintain a balance of power in the surgery context and adhere to practice protocols.
4. Maintain objectivity when treating another physician.
5. Stay empathetic, analyzing and recognizing any depreciation imposed by the patient in a therapeutic context.
6. Use mindfulness techniques (in particular the mindfulness-based stress reduction (MBSR) protocol) to manage stress and possible negative sensations and feelings in order to reduce pressure in anticipation of surgery.

### Limitations of This Research

Some limitations of this paper must be pointed out. The retrospective nature of this study could introduce data collection bias. It was not possible to design a ‘universal’ blepharoplasty/rhytidectomy/breast augmentation technique. Every patient presented with a unique set of problems that required precise anatomic diagnosis and an appropriately planned and individualized surgical repair. 

Although this study only considered interventions that had objective points of reference in the literature and that were carried out in the same way, it is also necessary to take into account the differences in the procedures and the type of patient, which may influence the complication rate. 

Furthermore, due to the absence of a VIP scale, we were forced to aggregate such different types of “VIPs” together (for example, a doctor and an international superstar).

In addition, the small patient numbers make true trends and statistical analysis problematic. The number of MDs/VIPs in this study is relatively small, and it would probably take a larger sample to show a statistical difference in some of the complication rates. However, it would take a very long time to study such a number of MDs/VIPs at the same institute.

This information could be valuable, however, in distinguishing potential trends for developing prospective studies in the future, for which strict observation and measurement criteria would be desirable.

The retrospective nature of this study meant that previous levels of burnout; the degrees of self-confidence, self-efficacy, self-esteem; performance anxiety; state anxiety; perceived stress level; the type of decision making; and perspectives of the surgeons were not taken into consideration.

Future studies will have to take these variables into consideration through experimental research designs that make use of self-reported questionnaires and ad hoc interviews for the medical operators involved.

Further studies with larger series, a prospective design, and control groups are necessary to confirm our results and definitively change the current perception of Murphy’s Law.

## 5. Conclusions

Our results demonstrate that there were no differences in the complications between MDs/VIPs vs. non-MD/VIPs after aesthetic surgery. Caring for MDs/VIPs creates unique and ethical challenges for surgeons. However, understanding these challenges can help the surgeon navigate through the barriers to care created by the status of the MD/VIP. Establishing a strong therapeutic alliance, maintaining professional boundaries, and treating the MDs/VIPs like any other patient are important principles to consider. Reviewing best practice guidelines and seeking consultation with peers can additionally be helpful to the surgeon. 

## Figures and Tables

**Table 1:** Demographic data of group 1 (VIPs and medical doctors) and group 2 (control group) who underwent upper blepharoplasty, facelift, or breast augmentation between January 2010 and October 2020.

	Blepharoplasty			Facelift			Breast Augmentation		
No. of Patients	Group 1 (n = 212)	Group 2 (n = 212)	*p*	Group 1 (n = 67)	Group 2 (n = 67)	*p*	Group 1 (n = 89)	Group 2 (n = 89)	*p*
Mean Patient Age at Surgery (Range, Years)	49.8 (36–76)	53.4 (38–78)	0.0000 0.0002	57.8 (48–79)	56.3 (46–75)	>0.05 0.1411	31.3 (18–56)	29.5 (19–47)	>0.05 0.3527
Sex									
Male	51	40	>0.05	11	25	>0.05	0	0	>0.05
Female	161	172	>0.05	56	42	>0.05	89	89	>0.05
BMI > 30									
Yes	34	37	0.0457	11	13	0.8702	17	23	0.6607
Smoking									
Yes	46	30	0.8296	17	19	>0.05	25	22	0.8610
Drugs									
Yes	43	52	>0.05	22	28	0.5634	11	21	0.5599

**Table 2:** (**A**) Postoperative complications noted amongst patients who underwent upper blepharoplasty. (**B**) Postoperative complications noted amongst patients who underwent facelift. (**C**) Postoperative complications noted amongst patients who underwent breast augmentation.

(A)				
Upper Eyelid Blepharoplasty Complication [6]	Incidence in the Literature	Our Experience		
		Group 1	Group 2	*p*
Symptomatic dry eye/exposure keratopathy [15]	16/124 (12.9%)	28 (13.2%)	23 (10.9%)	0.54
Conjunctival chemosis [15]	1/124 (0.8%)	2 (0.9%)	3 (1.4%)	0.9
Infection [16] (mean)	4/1332 (0.2%)	0	0	>0.5
Retrobulbar hemorrhage [17]	1/2000 (0.05%)	0	0	>0.5
Blindness [17]	1/10,000 (0.004%)	0	0	>0.5
Admission to outpatient clinic for medication (within 1 month after surgery, mean) [7]	1	2.3 (1–4)	1.2 (1–2)	<0.001
Length to follow-up (months; mean (range))		95 (32–124)	101 (34–128)	0.06
Time of operation (surgeon 1), minutes Mean (range) [18]	45 (30–60)	51 (40–61)	43 (34–58)	<0.001
Time of operation (surgeon 2), minutes Mean (range) [18]	45 (30–60)	53 (42–63)	45 (36–58)	<0.001
**(B)**				
**Facelift Complication**	**Incidence in the Literature (%)**	**Our Experience**		
		**Group 1** **(n = 67)**	**Group 2** **(n = 67)**	***p***
Hematoma minor [9]	108/4328 (2.50)	3 (4.47%)	5 (5.97%)	0.7
Hematoma major [9]	42/5719 (0.73)	1 (1.49%)	1 (1.49%)	>0.5
Cutaneous slough or necrosis [9]	50/7232 (0.69)	3 (4.47%)	2 (3.77%)	>0.5
Seroma [9]	12/823 (1.46)	2 (3.77%)	2 (3.77%)	>0.5
Nerve damage—temporary [9]	35/5081 (0.69)	1 (1.49%)	2 (3.77%)	>0.5
Nerve damage—permanent [9]	0/5638	0	0	>0.5
Infection [9]	22/4177 (0.53)	0	0	>0.5
Admission to outpatient clinic for medication (within 3 months) [10]	3	4.88 (4–8)	3.43 (3–5)	<0.001
Length to follow-up (months; mean (range))		67 (30–108)	89 (34–128)	<0.001
Time of operation (surgeon 1), minutes Mean, (range) [19,20]	90 (75–120)	103 (86–128)	97 (91–112)	<0.001
Time of operation (surgeon 2), minutes Mean (range) [19,20]	90 (75–120)	109 (93–129)	102 (92–116)	<0.001
**(C)**				
**Breast Augmentation Complication**	**Incidence in the Literature (mean, %)**	**Our Experience**		
		**Group 1 (n = 89)**	**Group 2 (*n* = 89)**	***p***
Infection [21]	16/2171 (0.73)	0	1 (1.12%)	>0.5
Implant rupture [21]	16/1186 (1.34)	1 (1.12%)	2 (2.24%)	>0.5
Hematoma [21]	37/1674 (2.21)	3 (3.37%)	3 (3.37%)	>0.5
Animation deformity [21]	5/426 (1.17)	5 (5.61%)	4 (4.49%)	>0.5
Rippling [21]	50/990 (5.05)	6 (6.74%)	5 (5.61%)	>0.5
Capsular contracture [21]	693/18,109 (3.82)	4 (4.49%)	3 (3.37%)	>0.5
Implant displacement [21]	20/1358 (1.47)	2 (2.24%)	2 (2.24%)	>0.5
Seroma [21]	10/10,501 (0.09)	1 (1.12%)	0	>0.5
Need for revision [21]	217/1975 (10.98)	12 (13.48%)	8 (8.98%)	0.35
Admission to outpatient clinic for medication (in 3 months, mean) [22]	3	5.1 (3–6)	3.2 (3–5)	<0.001
Length to follow-up (months; mean (range))		58 (26–104)	76 (32–118)	<0.001
Time of operation, minutes Mean (range) [13]	60 (45–90)	69 (53–78)	56 (45–68)	<0.001

## Data Availability

The original contributions presented in the study are included in the article, further inquiries can be directed to the corresponding authors.
